# Applying Multiple Machine Learning Models to Classify Mild Cognitive Impairment from Speech in Community-Dwelling Older Adults

**DOI:** 10.3390/jintelligence14020017

**Published:** 2026-01-26

**Authors:** Renqing Zhao, Zhiyuan Zhu, Zihui Huang

**Affiliations:** College of Physical Education, Yangzhou University, Yangzhou 225009, China

**Keywords:** mild cognitive impairment, speech classification features, machine learning, random forest, support vector machine

## Abstract

This study aims to develop effective screening tools for cognitive impairment by integrating optimised speech classification features with various machine learning models. A total of 65 patients diagnosed with early-stage Mild Cognitive Impairment (MCI) and 55 healthy controls (HCs) were included. Audio data were collected through a picture description task and processed using the Python-based Librosa library for speech feature extraction. Three machine learning models were constructed: the Random Forest (RF) and Support Vector Machine (SVM) models utilised speech classification features optimised via the Sequential Forward Selection (SFS) algorithm, while the Extreme Gradient Boosting (XGBoost) model was trained on preprocessed speech data. After parameter tuning, the Librosa library successfully extracted 41 speech classification features from all participants. The application of the SFS optimisation strategy and the use of preprocessed data significantly improved identification accuracy. The SVM model achieved an accuracy of 0.825 (AUC: 0.91), the RF model reached 0.88 (AUC: 0.86), and the XGBoost model attained 0.92 (AUC: 0.91). These results suggest that speech-based machine learning models markedly improve the accuracy of distinguishing MCI patients from healthy older adults, providing reliable support for early cognitive deficit identification.

## 1. Introduction

The global burden of Alzheimer’s Disease (AD) presents an urgent public health crisis (Mmadumbu et al., 2025; Scheltens et al., 2021). This situation becomes a particularly serious phenomenon in developing countries, such as China. By the end of 2024, the number of elderly people aged 60 and above had reached 310 million, accounting for 22.0% of the total national population (State Bureau of Statistics, 2025). The primary clinical manifestations of AD include progressive and significant decline in cognitive functions such as memory, language, thinking, and behavioural abilities, severely impacting the quality of life for patients and their families (Mmadumbu et al., 2025; Scheltens et al., 2021). However, there are currently no effective drugs that can completely cure AD (Antony et al., 2025). Therefore, the early identification and intervention of AD have become crucial public health issues. Research indicates that patients may develop Mild Cognitive Impairment (MCI) several years before clinical symptoms appear. Without early detection and intervention, approximately 10% to 15% of MCI patients progress to AD each year (Petersen, 2000). Worldwide, the prevalence of MCI among older adults is estimated to be approximately 23.7% and is influenced by various factors such as lower educational attainment, gender, geographic region, diagnostic tools, and levels of physical activity (Ismail & Morsy, 2025; Salari et al., 2025). This transitional status necessitates early and accurate detection, as timely intervention offers the most significant opportunity to potentially slow down or prevent disease progression (Ruzi et al., 2025).

Current established diagnostic approaches for AD and MCI, while providing high accuracy, are inherently resource-intensive, often invasive, and fundamentally lack the scalability required for population-level screening (Ruzi et al., 2025). Standard cognitive assessments, such as the mini-mental state examination (MMSE) and the Montreal cognitive assessment (MoCA), rely on standardised administration by trained clinicians. This dependency limits throughput, introduces potential variability, and restricts consistency across different clinical settings (X. Zhao et al., 2024). Beyond behavioural scales, high-fidelity imaging modalities are often used for diagnostic confirmation and disease staging. Fluorodeoxyglucose positron emission tomography scans are strongly correlated with patient symptoms and clinical severity and have been shown to predict conversion to AD dementia in MCI patients more effectively than conventional cerebrospinal fluid or magnetic resonance imaging (MRI) scans (Q. Zhao et al., 2023). Research evaluating the cost-effectiveness of adding to the standard clinical workup for suspected AD patients suggests that while it has high diagnostic accuracy, its benefits may be limited relative to the very high cost (Gerke et al., 2015). The resource demands of these methods contrast sharply with the logistical requirements of mass screening, rendering them unsuitable for initial, high-volume community assessment (Mayblyum et al., 2021; Oldan et al., 2021).

In light of the limitations of traditional methods, speech analysis has emerged as a promising, non-invasive biomarker for detecting cognitive decline. Speech acts as a multimodal output, encompassing both acoustic characteristics (paralinguistics) and complex linguistic structures (Devahi et al., 2025). MCI patients exhibit subtle changes in speech and language abilities at an early stage of AD (Yang et al., 2024). In recent years, research on early screening and risk assessment of cognitive impairment based on speech analysis technology has become an emerging hotspot due to its unique advantages. Human speech, as a complex product of cognitive behaviour, can sensitively reflect impairments in neural language networks, executive functions, and memory retrieval abilities associated with AD through subtle changes in acoustic, prosodic, lexical, and semantic features (Garcia-Gutierrez et al., 2024; Yang et al., 2024). Several lines of studies have preliminarily confirmed that analysing pause ratios, semantic density, and acoustic features in speech can effectively distinguish healthy older adults, MCI patients, and AD patients, demonstrating significant potential in terms of accuracy (Alsuhaibani et al., 2025; Garcia-Gutierrez et al., 2024). Automated speech and language analysis (ASLA) offers a highly appealing alternative due to its non-invasive, affordable, and exceptionally scalable (Perez-Toro et al., 2025). This fundamental shift in operational logistics makes ASLA an indispensable component of future public health infrastructure designed to meet the massive demographic challenges articulated by global dementia action plans.

With the rapid development of machine learning (ML), researchers can systematically extract and combine multi-dimensional speech biomarkers such as acoustic, prosodic, lexical, and semantic features to establish a more comprehensive characterisation system for cognitive dysfunction (Saleem et al., 2022; Toumaj et al., 2024). Computational paralinguistics, which derives features directly from audio signals, provides significant logistical advantages (Haider et al., 2020) by capturing voice quality, rhythm, and articulation. Key features include spectro-temporal measures such as mel-frequency cepstral coefficients (MFCCs), temporal perturbations like jitter (frequency variation) and shimmer (amplitude variation), and voice quality metrics such as the harmonics-to-noise ratio (HNR) (Shah et al., 2021). Furthermore, leveraging refined audio data—such as those processed with the speech filing system (SFS)—classical ML algorithms like random forest (RF) and support vector machines (SVM) have demonstrated improved classification of cognitive impairment (Hason & Krishnan, 2022; Remya et al., 2025; Zhang et al., 2024). However, existing clinical research in this domain predominantly relies on data from Western elderly populations, with limited studies on Chinese older adults. To address this gap, this study investigates speech features in Chinese older adults, applying various ML models to analyse audio biomarkers for early detection of MCI. By evaluating and comparing the classification performance across models, this research aims to validate the efficacy of speech analysis as a viable tool for early screening and identification of cognitive dysfunction.

## 2. Materials and Methods

### 2.1. Participants

Four hundred and fifty native Chinese speakers aged 60–80 years were recruited from local communities. Each participant completed two questionnaires: one collected baseline information such as medical history, income, and chronic diseases, while the other assessed cognitive status. After the initial assessment, 108 participants were excluded due to medical conditions, visible or hearing problems, or lack of interest. Of the remaining individuals, 67 met the criteria for Mild Cognitive Impairment (MCI) and 275 were classified as Healthy Controls (HCs). To balance the groups, a random subset of HCs was excluded, leaving 65 HCs for language testing. Following attrition, including loss of contact and withdrawal of consent, the final analytic sample consisted of 120 participants: 55 HCs and 65 individuals with MCI. MCI participants were diagnosed by board-certified neurologists using the Montreal Cognitive Assessment (MoCA), with a cutoff score below 26 (Nasreddine et al., 2005). Diagnoses were further confirmed through comprehensive neuropsychiatric and neuropsychological evaluations (Garcia-Cordero et al., 2019). Participants with MCI had no history of other neurological disorders or primary language deficits. Linguistic function was verified through neuropsychological interviews and qualitative assessment of conversational speech. Healthy controls were cognitively normal, functionally independent, and reported no history of neuropsychiatric illness or substance abuse. All participants had normal hearing or corrected-to-normal hearing, verified through a formal functional hearing survey. The two groups (MCI and HCs) were matched for sex, age, and years of education. Detailed demographic and neuropsychological characteristics are presented in Table 1. All participants provided written informed consent, which was documented by a member of the research team. The study was conducted in compliance with the Declaration of Helsinki and approved by the Ethics Committee of Yangzhou University.

### 2.2. Speech Elicitation and Transcription

Speech samples were collected using a structured yet naturalistic elicitation task based on the well-established “Cookie-Theft” picture from the Boston Diagnostic Aphasia Examination. This task is widely used in neuropsychological assessment because it reliably evokes spontaneous, connected speech while placing minimal cognitive burden on participants. During the interview, each participant was shown the picture and provided with a simple, open-ended prompt instructing them to describe everything they observed occurring in the scene. This design encourages individuals to generate continuous narrative speech, allowing for the capture of linguistic, semantic, and prosodic features that may be sensitive to early cognitive decline. The participants’ verbal responses were recorded in their entirety to preserve the full acoustic richness of the speech signal. This included not only lexical content but also prosodic contours, temporal characteristics, hesitation patterns, and other paralinguistic cues that often reflect subtle cognitive or motor changes. Recording conditions were kept consistent across participants to minimise variability related to environmental noise or equipment differences. Following data collection, all audio recordings underwent meticulous transcription using the CHAT (Codes for the Human Analysis of Transcripts) format, a standardised system widely adopted in language and speech research.

### 2.3. Audio Feature Extraction and Integration

Audio features were extracted from speech recordings of individuals with MCI and HC using the Librosa library (version 0.10.1) in Python (version 3.8) adjusted to account for the characteristics of Chinese pronunciation. The dataset comprised spontaneous speech samples (e.g., picture descriptions) from MCI patients and age-matched controls, stored in WAV format with sampling rates of 16–44.1 kHz. Features focused on acoustic and paralinguistic markers, including MFCCs, chroma, spectral contrast, tonnetz, zero-crossing rate (ZCR), root-mean-square (RMS) energy, and pitch, which capture spectral, harmonic, and prosodic alterations associated with MCI-related cognitive decline (Filiou et al., 2019; Jafari et al., 2025). Features were extracted at the utterance level, normalised using Z-score standardisation to reduce variability across acoustic scales and recording conditions, and subsequently aggregated by computing global statistical descriptors—such as mean, standard deviation, skewness, and kurtosis. This process converted variable-length temporal sequences into fixed-dimension feature vectors suitable for input into machine-learning models for MCI classification. Dependencies included NumPy (v1.24.4) for array handling and SciPy (v1.13.1) for statistical operations. The features were then input to classifiers (e.g., random forests or support vector machines) for binary MCI vs. HCs classification. This method supports non-invasive MCI screening via speech biomarkers. Code is available upon request, adhering to Librosa documentation for reproducibility.

### 2.4. Data Processing and Model Construction

SFS was applied to the full acoustic and paralinguistic feature set. The procedure was performed on the training data using the F1-score as the selection criterion for the SVM and RF classifiers. Starting from an empty set, SFS iteratively added the most discriminative features based on cross-validation accuracy, with subsets evaluated using an RBF kernel and hyperparameters tuned via grid search. SFS was executed independently within each fold of cross-validation. RF and SVM models were trained and tested on both the full feature set and the SFS-selected subset, allowing performance to be compared within a consistent, systematically derived feature space. Subsequently, SVM and RF models were developed using a dataset comprising 120 participants. The participants were divided into an 80% training set and a 20% independent holdout test set, with each subject assigned exclusively to one group to prevent data leakage. Normalisation parameters were fitted exclusively on the training data and applied to the corresponding test folds to prevent data leakage. Feature extraction and hyperparameter tuning were performed within the standard 10-fold cross-validation (CV) framework applied to the training set, ensuring unbiased model evaluation. Model stability was further assessed using 1000 bootstrap resamples. Model performance was evaluated using five standard classification metrics: accuracy, precision, recall, F1-score, and AUC-ROC. These metrics were calculated as the average across the 10-fold cross-validation folds within the training set. Final model performance was then assessed on the independent holdout test set using the same evaluation metrics. For XGBoost model training, a comprehensive data preprocessing pipeline was implemented. The procedure included normalising feature distributions, encoding categorical variables, and removing outliers. Subsequently, the model was fitted with hyperparameters optimized through grid search and early stopping to prevent overfitting. XGBoost analyses were conducted on the MCI and HCs groups, yielding comparative performance metrics and feature importance rankings to identify key acoustic biomarkers for cognitive impairment detection.

### 2.5. Statistical Analyses

A comprehensive statistical analysis was conducted using the Python SciPy library to ensure rigorous evaluation of the extracted acoustic features. The analytical workflow followed a structured decision framework designed to assess distributional properties, variance characteristics, and between-group differences. Initially, the Shapiro–Wilk test was applied to each acoustic parameter to determine whether the data conformed to a normal distribution. For features demonstrating normality, Levene’s test was subsequently performed to examine the homogeneity of variance between the healthy control and cognitively impaired groups. Parameters satisfying both assumptions were analysed using independent samples *t*-tests to identify statistically significant group differences. For features that violated normality or exhibited unequal variances, the non-parametric Mann–Whitney U test was employed as a robust alternative, ensuring that inference remained valid despite distributional irregularities. In addition to acoustic measures, demographic and clinical variables—including age, years of education, and MoCA scores—were compared across groups using one-way ANOVA for normally distributed continuous variables, with post hoc Tukey tests applied where appropriate. Non-normally distributed variables were analysed using Kruskal–Wallis tests. Categorical variables, such as sex, were evaluated using chi-square tests. All statistical tests were two-tailed, with a significance threshold of α = 0.05. This multi-step analytical strategy ensured that each feature was assessed using the most appropriate inferential method, thereby enhancing the reliability and interpretability of the study’s findings.

## 3. Results

### 3.1. Demographic Data

Table 1 summarises the demographic and clinical characteristics of the study participants. The MCI and healthy control groups were closely matched in age, indicating that observed differences in speech-based measures were unlikely to be confounded by age-related variability. The sex distribution was also comparable between groups, with no statistically significant difference in the proportion of male and female participants. As expected and consistent with the diagnostic criteria for MCI, the groups differed significantly in their MoCA scores, with the MCI group scoring substantially lower than the HC group. This disparity in MoCA performance confirms the presence of measurable cognitive impairment in the MCI cohort while underscoring the cognitive integrity of the control participants, thereby supporting the validity of the group classifications used in this study.

### 3.2. Processing and Analysis of Raw Audio Data

**Figure 1:**
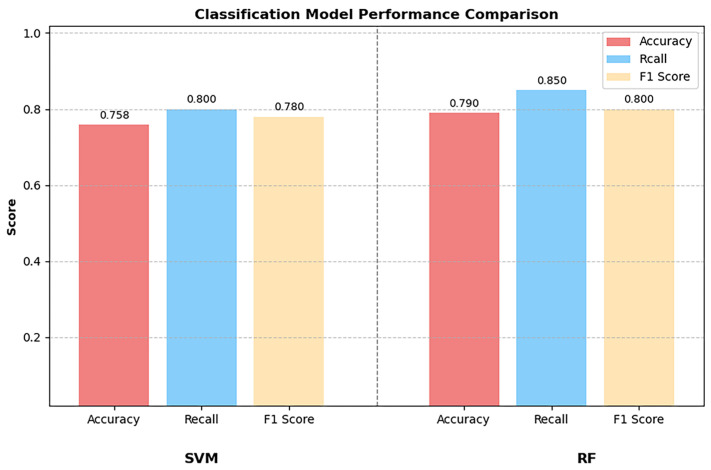
RF and SVM Classification Models. RF, random forest; SVM, support vector machine.

**Figure 2:**
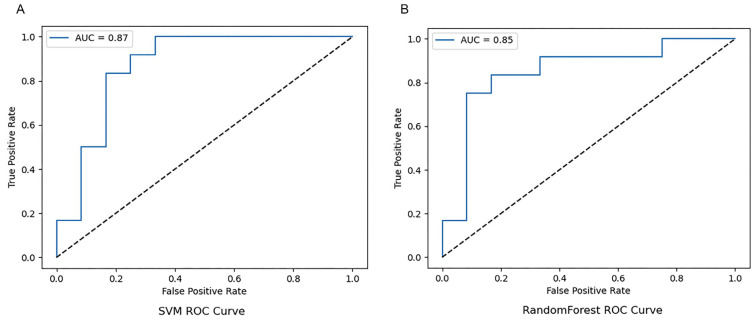
Receiver Operating Characteristic (ROC) Curve. (**A**) RF, random forest; (**B**) SVM, support vector machine.

Each participant in this study provided an independent piece of speech data. These data were collected through a standardised speech task: picture description. First, the efficient reading capabilities of Soundfile and the audio splicing function of librosa were used to process the raw speech data. Subsequently, the librosa library was employed to extract features closely related to speech classification, such as MFCC, spectral centroid, and spectral bandwidth. These features served as key inputs for subsequent model training. In the model validation phase, two classic machine learning models—RF and SVM—were used. Accuracy, F1-score, and AUC (Area Under the ROC Curve) were adopted as core evaluation metrics to assess the data classification performance. The results showed that the identification accuracy of the SVM model was 0.758 (AUC: 0.87), while the RF model achieved a significantly higher identification accuracy of 0.79 (AUC: 0.85) (Figure 1 and Figure 2).

### 3.3. SFS-Optimised Speech Classification Features and Analysis of RF and SVM Models

**Figure 3:**
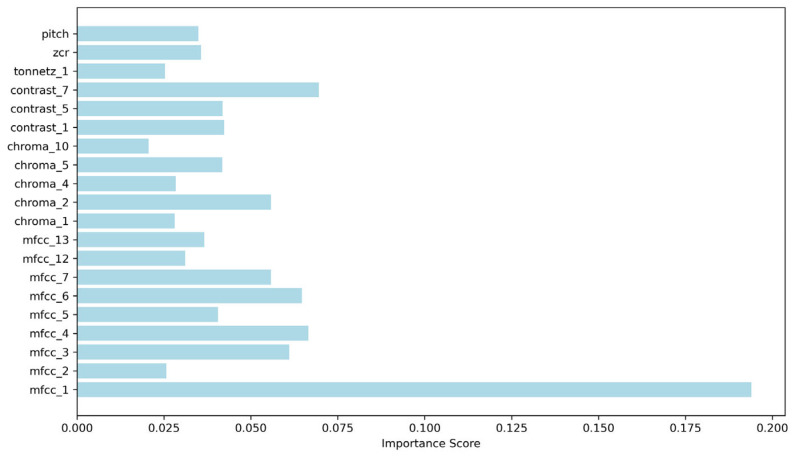
Optimal Audio Classification Features Identified by Sequential Forward Selection (SFS).

**Figure 4:**
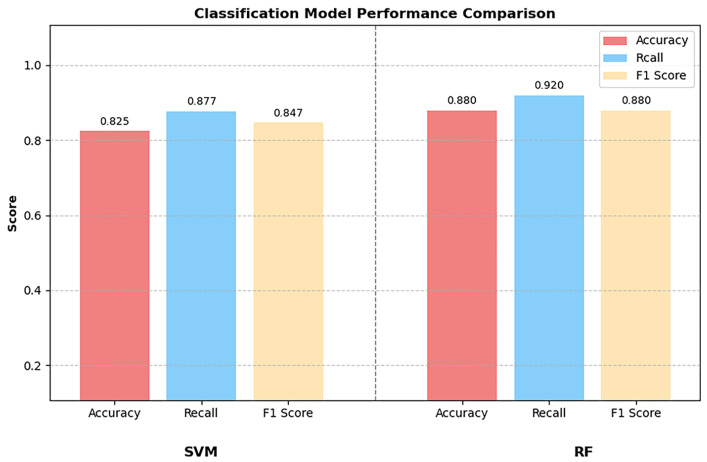
RF and SVM Classification models with SFS-processed data. RF, random forest; SVM, support vector machine; SFS, sequential forward selection.

**Figure 5:**
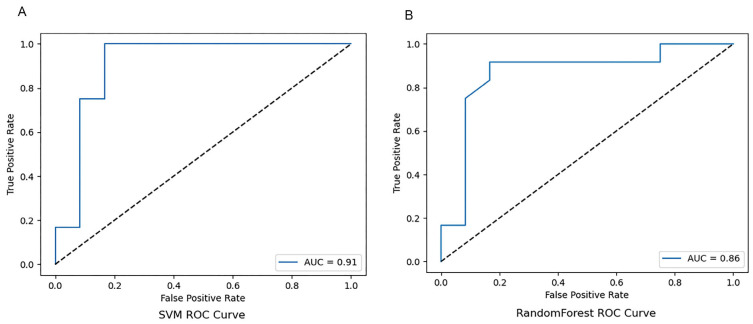
Receiver Operating Characteristic (ROC) Curve with SFS-processed data. (**A**) RF, random forest; (**B**) SVM, support vector machine.

To further improve model performance, this study introduced the SFS algorithm for optimising speech classification features. By iteratively selecting the most discriminative feature subsets, this method achieved three optimisation objectives: enhancing the accuracy of model classification, strengthening the relevance between features and the classification task, and optimising feature dimensions tailored to the characteristics of small-scale datasets to improve model interpretability. Results of feature optimisation showed that the 20 core speech features selected by the SFS algorithm had the highest contribution rate, forming the optimal feature subset (Figure 3). Re-training and evaluating the models based on this optimised feature set revealed that the RF model performance was significantly improved, with a classification accuracy of 0.88 and an AUC value of 0.86 (Figure 4 and Figure 5). In contrast, the accuracy of 0.825 predicted by the SVM model performance remained unchanged, only with an improved AUC of 0.91 (Figure 4 and Figure 5). These findings confirm that feature optimisation can significantly improve the accuracy of cognitive impairment identification, while also revealing that different machine learning models exhibit marked differences in their sensitivity to feature optimisation.

### 3.4. XGBoost Model Evaluation

**Figure 6:**
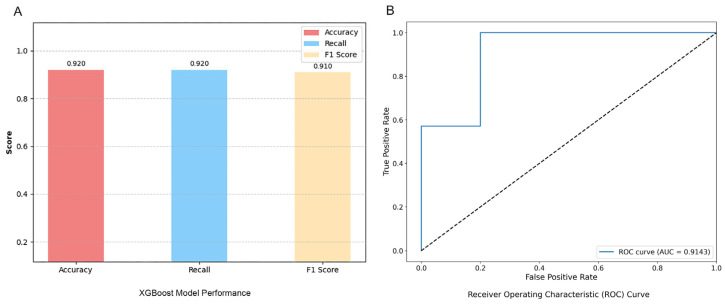
XGBoost Classification Models (**A**) and Receiver Operating Characteristic (ROC) Curve (**B**).

Additionally, for speech classification features, this study adopted a preprocessing strategy tailored to the XGBoost model. Key steps, such as standardising feature scale values, were implemented to optimise the distribution of input data, providing a more suitable feature matrix for model training. The XGBoost model was constructed and evaluated based on the preprocessed feature set, yielding an identification accuracy of 0.92 and an AUC value of 0.91 (Figure 6). It appeared that the XGBoost model with targeted preprocessing might outperform the RF and SVM models in identifying MCI from HCs.

## 4. Discussion

This study provides experimental evidence demonstrating that machine learning models trained on speech data collected from community-dwelling older adults exhibit strong effectiveness in the classification of MCI. By leveraging non-invasive, low-cost, and easily deployable audio-based assessments, the proposed approach offers a promising avenue for identifying individuals at early stages of cognitive decline. Early detection is particularly critical given the progressive nature of AD, where timely intervention may help delay or mitigate the transition from MCI to dementia. The findings of this study therefore contribute to the growing body of research supporting speech-based digital biomarkers as practical tools for large-scale cognitive screening.

The rapid advancement of non-invasive diagnostic technologies has highlighted the substantial potential of speech analysis in the early detection of cognitive impairment. Speech is a complex cognitive-motor behaviour that integrates linguistic, semantic, executive, and motor processes. As such, subtle disruptions in speech patterns often emerge before overt clinical symptoms become apparent. Compared with neuroimaging or biomarker-based assessments, speech data can be collected quickly, inexpensively, and with high patient compliance, making it particularly suitable for community and home-based screening. Prior studies have provided compelling evidence supporting the utility of speech-derived features as supplementary indicators for clinical diagnosis (Alsuhaibani et al., 2025; Garcia-Gutierrez et al., 2024). These studies collectively underscore the feasibility of integrating speech analytics into routine cognitive health monitoring.

In the broader context of neurodegenerative disease screening, alterations in speech signals have been shown to correlate closely with disease progression. Tracy et al. (Tracy et al., 2020) demonstrated that speech analysis models built using traditional machine learning algorithms could effectively differentiate Parkinson’s disease (PD) patients from healthy controls, achieving an AUC of 0.88. This work illustrates how acoustic and prosodic features can capture early motor and cognitive changes associated with PD, offering a non-invasive alternative to conventional diagnostic tools. Extending this to geriatric health evaluations, Rosen-Lang et al. (Rosen-Lang et al., 2024) employed sophisticated acoustic feature extraction methods to uncover significant disparities in metrics like peak volume ratio and pause variability between older adults exhibiting high versus low frailty. These findings affirm the practical viability of speech analytics in categorising cognitive decline among the elderly, introducing an innovative lens for managing mental health in ageing populations. Such insights emphasise how speech can serve as a window into neurological health, capturing subtle deteriorations that might elude traditional assessments like questionnaires or physical exams.

Further supporting this line of evidence, researchers have combined automatic speech analysis with machine learning algorithms to classify healthy older adults, MCI patients, and AD patients, achieving an average recognition accuracy of 82.0% (5). Such results highlight the potential of speech-derived biomarkers for stratified screening across different stages of cognitive impairment. Compared with these prior studies, the speech analysis model developed in the present work demonstrated superior performance, suggesting that the integration of optimised feature selection and model tuning can yield meaningful improvements in classification accuracy.

More directly pertinent to our work, prior investigations have successfully employed automatic speech analysis combined with machine learning to distinguish among healthy elderly individuals, those with MCI, and those diagnosed with AD. One such study reported an average classification accuracy of 82.0% across these three groups (Konig et al., 2015), establishing a foundational benchmark for stratified cognitive screening. Notably, the model developed in our study outperformed this baseline, suggesting that methodological refinements—particularly in feature selection and algorithm optimisation—can yield meaningful gains in diagnostic precision.

To enhance the optimisation of speech features for cognitive impairment classification, this study employed the SFS algorithm to identify key feature subsets, followed by comparative evaluation of RF and SVM models. This methodological framework offers several advantages with strong clinical relevance. First, SFS-based feature selection effectively distilled 20 core features from a high-dimensional dataset, thereby improving model efficiency, reducing overfitting risk, and enhancing interpretability. In clinical contexts, the identification of well-defined speech biomarkers—such as specific prosodic, temporal, or spectral characteristics—can deepen understanding of disease manifestations and provide valuable insights for exploring underlying neuropathological mechanisms (Kuchibhotla et al., 2016; Ververidis & Kotropoulos, 2005). Feature interpretability is particularly important for clinical adoption, as clinicians increasingly seek transparent and explainable AI tools that complement traditional diagnostic reasoning. Secondly, our results illuminated a marked improvement in SVM performance post-feature engineering, laying the groundwork for crafting precise, non-invasive, and economical instruments for preliminary cognitive screening. This is particularly pertinent in resource-limited settings, where advanced neuroimaging or biomarker assays are infeasible. By prioritising features that maximise discriminative ability, we mitigate overfitting risks and enhance generalizability across diverse populations, including those with varying dialects or accents. Furthermore, the comparative edge of SVM over RF in our optimised framework highlights the importance of kernel-based methods in handling non-linear relationships inherent in speech data, which often exhibit complex interactions due to individual variability in vocal production.

Beyond RF and SVM models, this study also explored the application of alternative machine learning approaches, with particular emphasis on the XGBoost model. XGBoost has gained widespread recognition for its strong performance in structured data analysis, owing to its ability to integrate comprehensive data preprocessing, regularisation strategies, and optimised tree-based learning. Prior research has demonstrated that XGBoost can effectively capture nonlinear relationships and interactions among speech features, making it well-suited for detecting subtle cognitive changes (Chau & Chau, 2024). In this study, XGBoost exhibited notable strengths in classifying cognitive impairment, outperforming unoptimized RF and SVM models. These findings underscore the value of incorporating advanced ensemble learning techniques into speech-based diagnostic pipelines, particularly when dealing with heterogeneous and high-dimensional feature spaces.

The practical implications of this work are substantial. As populations age globally, scalable and accessible screening tools for cognitive impairment are urgently needed. Traditional clinical assessments often require specialised personnel, are time-consuming, and may not be feasible for routine monitoring. In contrast, speech-based machine learning models can be deployed through smartphones, telemedicine platforms, or community health programs, enabling continuous and unobtrusive monitoring of cognitive health. Such tools could support early identification of at-risk individuals, guide timely referrals for clinical evaluation, and facilitate personalised intervention strategies aimed at slowing cognitive decline. Collectively, this study demonstrates that machine learning models trained on speech data offer a powerful, non-invasive, and practical approach for classifying MCI in community settings. By integrating optimised feature selection with advanced machine learning algorithms, the proposed framework provides a robust foundation for developing scalable early screening tools. As digital health technologies continue to evolve, speech-based cognitive assessment holds significant promise for transforming early detection and intervention strategies in ageing populations.

## 5. Strengths and Limitations

One of the strengths of this study is evaluate the diagnostic utility of speech-based biomarkers as a scalable, non-invasive tool for early cognitive impairment screening. However, several limitations of this study should be acknowledged. First, the model selection was restricted to RF, SVM, and XGBoost, without incorporating more advanced approaches such as deep learning, which may capture subtler acoustic patterns and potentially improve MCI classification. Second, the relatively small clinical sample size may limit the representativeness and generalizability of the results. In addition, the study did not comprehensively examine the clinical significance of the identified core speech features or their potential associations with underlying pathophysiological mechanisms of cognitive impairment. This gap constrains both the interpretability of the findings and the translational potential of the technology in real-world clinical contexts. Future research should address these limitations by increasing sample size, evaluating more sophisticated modelling strategies, and integrating neurocognitive perspectives to enhance the clinical relevance and biological plausibility of speech-based MCI classification.

## Figures and Tables

**Table 1:** Demographic and Neuropsychological Characteristics of Participants with MCI and HCs.

Variables	MCI (*n* = 65)	HCs (*n* = 55)	Total (*n* = 120)	Statistical Value	*p* Value
Age, years	68.1 (10.5)	66.8 (9.7)	67.4 (10.1)	t = −0.70	0.484
Educational level, years	13.7 (5.2)	15.7 (4.5)	14.0 (4.9)	t = −0.68	0.497
Sex, n (Male/Female)	36/29	21/34	57/63	χ^2^ = 3.534	0.060
MoCA score	23.6 (1.8)	28.1 (1.2)	25.7 (1.5)	−16.32	<0.001

Notes: MoCA, Montreal Cognitive Assessment; MCI, mild cognitive impairment; HCs, healthy controls. Data were represented as mean (std).

## Data Availability

The original contributions presented in this study are included in the article. The corresponding author will provide the necessary data supporting the findings of this study upon a reasonable request. The authors are accountable for ensuring the continued availability of the data.

## References

[B1-jintelligence-14-00017] Alsuhaibani M., Pourramezan Fard A., Sun J., Far Poor F., Pressman P. S., Mahoor M. H. (2025). A review of machine learning approaches for non-invasive cognitive impairment detection. IEEE Access.

[B2-jintelligence-14-00017] Antony D., Sheth P., Swenson A., Smoller C., Maguire K., Grossberg G. (2025). Recent advances in Alzheimer’s disease therapy: Clinical trials and literature review of novel enzyme inhibitors targeting amyloid precursor protein. Expert Opinion Pharmacotherapy.

[B3-jintelligence-14-00017] Chau H.-H., Chau Y. (2024). Audio-based classification of mild cognitive impairment using XGBoost. 2024 IEEE 6th Eurasia Conference on Biomedical Engineering, Healthcare and Sustainability (ECBIOS).

[B4-jintelligence-14-00017] Devahi A. S. E. S., Sangha S. S., Priyadarshinee P., Thilakan J., Tan I. F. X., Clarke C. J., Lon S. K., Quin Y. W., Jer-Ming C. (2025). Linguistic and audio embedding-based machine learning for Alzheimer’s dementia and mild cognitive impairment detection: Insights from the PROCESS challenge. arXiv.

[B5-jintelligence-14-00017] Filiou R.-P., Bier N., Slegers A., Houzé B., Belchior P., Brambati S. M. (2019). Connected speech assessment in the early detection of Alzheimer’s disease and mild cognitive impairment: A scoping review. Aphasiology.

[B6-jintelligence-14-00017] Garcia-Cordero I., Sedeño L., Babino A., Dottori M., Melloni M., Martorell Caro M., Sigman M., Herrera E., Manes F., García A. M., Ibáñez A. (2019). Explicit and implicit monitoring in neurodegeneration and stroke. Scientific Reports.

[B7-jintelligence-14-00017] Garcia-Gutierrez F., Alegret M., Marquié M., Muñoz N., Ortega G., Cano A., De Rojas I., García-González P., Olivé C., Puerta R., García-Sanchez A. (2024). Unveiling the sound of the cognitive status: Machine Learning-based speech analysis in the Alzheimer’s disease spectrum. Alzheimer’s Research Therapy.

[B8-jintelligence-14-00017] Gerke O., Hermansson R., Hess S., Schifter S., Vach W., Hoilund-Carlsen P. F. (2015). Cost-effectiveness of PET and PET/computed tomography: A systematic review. PET Clinics.

[B9-jintelligence-14-00017] Haider F., de la Fuente S., Luz S. (2020). An assessment of paralinguistic acoustic features for detection of Alzheimer’s dementia in spontaneous speech. IEEE Journal of Selected Topics in Signal Processing.

[B10-jintelligence-14-00017] Hason L., Krishnan S. (2022). Spontaneous speech feature analysis for Alzheimer’s disease screening using a random forest classifier. Frontiers Digital Health.

[B11-jintelligence-14-00017] Ismail A. M. A., Morsy M. M. (2025). Effect of Baduanjin exercise on lipid profile, blood pressure, and thyroid-stimulating hormone in elderly with subclinical hypothyroidism and mild cognitive impairment: A randomized-controlled trial in women. Geriatric Nursing.

[B12-jintelligence-14-00017] Jafari Z., Andrew M. K., Rockwood K. J. (2025). Diagnostic utility of speech-based biomarkers in mild cognitive impairment: A systematic review and meta-analysis. Age Ageing.

[B13-jintelligence-14-00017] Konig A., Satt A., Sorin A., Hoory R., Toledo-Ronen O., Derreumaux A., Manera V., Verhey F., Aalten P., Robert P. H., David R. (2015). Automatic speech analysis for the assessment of patients with predementia and Alzheimer’s disease. Alzheimers Dementia.

[B14-jintelligence-14-00017] Kuchibhotla S., Vankayalapati H. D., Anne K. R. (2016). An optimal two stage feature selection for speech emotion recognition using acoustic features. International Journal of Speech Technology.

[B15-jintelligence-14-00017] Mayblyum D. V., Becker J. A., Jacobs H. I., Buckley R. F., Schultz A. P., Sepulcre J., Sanchez J. S., Rubinstein Z. B., Katz S. R., Moody K. A., Vannini P. (2021). Comparing PET and MRI biomarkers predicting cognitive decline in preclinical Alzheimer disease. Neurology.

[B16-jintelligence-14-00017] Mmadumbu A. C., Saeed F., Ghaleb F., Qasem S. N. (2025). Early detection of Alzheimer’s disease using deep learning methods. Alzheimers Dementia.

[B17-jintelligence-14-00017] Nasreddine Z. S., Phillips N. A., Bédirian V., Charbonneau S., Whitehead V., Collin I., Cummings J. L., Chertkow H. (2005). The montreal cognitive assessment, MoCA: A brief screening tool for mild cognitive impairment. Journal American Geriatrics Society.

[B18-jintelligence-14-00017] Oldan J. D., Jewells V. L., Pieper B., Wong T. Z. (2021). Complete evaluation of dementia: PET and MRI correlation and diagnosis for the neuroradiologist. AJNR American Journal Neuroradiology.

[B19-jintelligence-14-00017] Perez-Toro P. A., J Ferrante F., Pérez G., Tee B. L., de Leon J., Nöth E., Schuster M., Maier A., Slachevsky A., Gorno-Tempini M. L., Ibáñez A. (2025). Automated speech markers of Alzheimer dementia: Test of cross-linguistic generalizability. Journal of Medical Internet Research.

[B20-jintelligence-14-00017] Petersen R. C. (2000). Mild cognitive impairment: Transition between aging and Alzheimer’s disease. Neurologia.

[B21-jintelligence-14-00017] Remya M. S., Raman R., Sankaran R., Namboodiri V., Nedungadi P. (2025). Artificial intelligence for speech classification and enhancement of speech and language disorders: Techniques, applications, and future directions. IEEE Access.

[B22-jintelligence-14-00017] Rosen-Lang Y., Zoubi S., Cialic R., Orenstein T. (2024). Using voice biomarkers for frailty classification. Geroscience.

[B23-jintelligence-14-00017] Ruzi R., Pan Y., Ng M. L., Su R., Wang L., Dang J., Liu L., Yan N. (2025). A speech-based mobile screening tool for mild cognitive impairment: Technical performance and user engagement evaluation. Bioengineering.

[B24-jintelligence-14-00017] Salari N., Lotfi F., Abdolmaleki A., Heidarian P., Rasoulpoor S., Fazeli J., Najafi H., Mohammadi M. (2025). The global prevalence of mild cognitive impairment in geriatric population with emphasis on influential factors: A systematic review and meta-analysis. BMC Geriatrics.

[B25-jintelligence-14-00017] Saleem T. J., Zahra S. R., Wu F., Alwakeel A., Alwakeel M., Jeribi F., Hijji M. (2022). Deep learning-based diagnosis of Alzheimer’s disease. Journal of Personalized Medicine.

[B26-jintelligence-14-00017] Scheltens P., De Strooper B., Kivipelto M., Holstege H., Chételat G., Teunissen C. E., Cummings J., van der Flier W. M. (2021). Alzheimer’s disease. The Lancet.

[B27-jintelligence-14-00017] Shah Z., Sawalha J., Tasnim M., Qi S.-A., Stroulia E., Greiner R. (2021). Learning language and acoustic models for identifying Alzheimer’s dementia from speech. Frontiers in Computer Science.

[B28-jintelligence-14-00017] State Bureau of Statistics (2025). Statistical bulletin on the national economic and social development of the People’s Republic of China in 2024.

[B29-jintelligence-14-00017] Toumaj S., Heidari A., Shahhosseini R., Jafari Navimipour N. (2024). Applications of deep learning in Alzheimer’s disease: A systematic literature review of current trends, methodologies, challenges, innovations, and future directions. Artificial Intelligence Review.

[B30-jintelligence-14-00017] Tracy J. M., Ozkanca Y., Atkins D. C., Hosseini Ghomi R. (2020). Investigating voice as a biomarker: Deep phenotyping methods for early detection of Parkinson’s disease. Journal of Biomedical Informatics.

[B31-jintelligence-14-00017] Ververidis D., Kotropoulos C. (2005). Sequential forward feature selection with low computational cost. 2005 13th European Signal Processing Conference.

[B32-jintelligence-14-00017] Yang X., Hong K., Zhang D., Wang K. (2024). Early diagnosis of Alzheimer’s Disease based on multi-attention mechanism. PLoS ONE.

[B33-jintelligence-14-00017] Zhang C., Guo W., Dai H. (2024). Automatic detection of mild cognitive impairment using high-dimensional acoustic features in spontaneous speech. arXiv.

[B34-jintelligence-14-00017] Zhao Q., Du X., Chen W., Zhang T., Xu Z. (2023). Advances in diagnosing mild cognitive impairment and Alzheimer’s disease using (11)C-PIB- PET/CT and common neuropsychological tests. Frontiers in Neuroscience.

[B35-jintelligence-14-00017] Zhao X., Wen H., Xu G., Pang T., Zhang Y., He X., Hu R., Yan M., Chen C., Wu X., Xu X. (2024). Validity, feasibility, and effectiveness of a voice-recognition based digital cognitive screener for dementia and mild cognitive impairment in community-dwelling older Chinese adults: A large-scale implementation study. Alzheimers Dementia.

